# Health system guidance appraisal—concept evaluation and usability testing

**DOI:** 10.1186/s13012-015-0365-3

**Published:** 2016-01-05

**Authors:** Denis E. Ako-Arrey, Melissa C. Brouwers, John N. Lavis, Mita K. Giacomini, on behalf of the AGREE HS Team

**Affiliations:** 1 McMaster University, Juravinski Hospital Site, G Wing, 2nd Floor, Room 207, 711 Concession Street, Hamilton, Ontario L8V 1C3 Canada; 2 McMaster University, MML-417, 1280 Main St. West, Hamilton, ON L8S 4L6 Canada; 3 McMaster University, CRL-218, 1280 Main Street West, Hamilton, ON L8S 4K1 Canada

**Keywords:** Health system guidance, Guidance development, Guidance appraisal, Guidance reporting, Health system challenges, Health system arrangements, AGREE-HS

## Abstract

**Background:**

Health system guidance (HSG) provides recommendations aimed to address health system challenges. However, there is a paucity of methods to direct, appraise, and report HSG. Earlier research identified 30 candidate criteria (concepts) that can be used to evaluate the quality of HSG and guide development and reporting requirements. The objective of this paper was to describe two studies aimed at evaluating the importance of these 30 criteria, design a draft HSG appraisal tool, and test its usability.

**Methods:**

This study involved a two-step survey process. In step 1, respondents rated the 30 concepts for appropriateness to, relevance to, and priority for health system decisions and HSG. This led to a draft tool. In step 2, respondents reviewed HSG documents, appraised them using the tool, and answered a series of questions. Descriptive analyses were computed.

**Results:**

Fifty participants were invited in step 1, and we had a response rate of 82 %. The mean response rates for each concept within each survey question were universally favorable. There was also an overall agreement about the need for a high-quality tool to systematically direct the development, appraisal, and reporting of HSG. Qualitative feedback and a consensus process by the team led to refinements to some of the concepts and the creation of a beta (draft) version of the HSG tool. In step 2, 35 participants were invited and we had a response rate of 74 %. Exploratory analyses showed that the quality of the HSGs reviewed varied as a function of the HSG item and the specific document assessed. A favorable consensus was reached with participants agreeing that the HSG items were easy to understand and easy to apply. Moreover, the overall agreement was high for the usability of the tool to systematically direct the development (85 %), appraisal (92 %), and reporting (81 %) of HSG. From this process, version 1.0 of the HSG appraisal tool was generated complete with 32 items (and their descriptions) and 4 domains.

**Conclusions:**

The final tool, named the Appraisal of Guidelines for Research and Evaluation for Health Systems (AGREE-HS) (version 1), defines expectations of HSG and facilitates informed decisions among policymakers on health system delivery, financial, and governance arrangements.

**Electronic supplementary material:**

The online version of this article (doi:10.1186/s13012-015-0365-3) contains supplementary material, which is available to authorized users.

## Background

Defining features of a health system can have a significant and direct impact on the health of individuals and communities [1]. By definition, a health system refers to governance arrangements (e.g., policy, organizational, or professional authority), financial arrangements (e.g., financing, funding, remuneration, or incentives), and delivery arrangements (e.g., to whom care is provided, by whom care is provided, or where care is provided) for health care and population health services and the broader context in which they are negotiated, implemented, and reformed [2–4]. Strengthening health systems is increasingly seen as a foundation for optimizing and maintaining improvements in population health outcomes, as well as in improving the patient experience and keeping per capita costs manageable [5–8].

The achievement of health goals in several countries and regions has been hindered by a variety of challenges ranging from weak and dysfunctional health system features like existing delivery, financial, and governance arrangements [3, 9], through influences on the policy process that compromise efforts like institutions, interests, and ideas [10], to context-specific features (political, social, cultural, and economic) that run counter to goals [11, 12]. Improving the suitability of health systems to deliver health care and public health interventions is, therefore, an essential quality goal. To this end, there has been an international effort among health system research and leader communities to leverage evidence, best practices, and transparent and systematic methods to strengthen health systems. Health system guidance (HSG) are knowledge tools that can be used to achieve this complex goal.

HSGs are systematically developed statements produced at global, national, and regional levels (e.g., by the World Health Organization, ministries and departments of health, and special committees supporting ministries and departments of health) that provide possible courses of action to address these challenges and thereby strengthen health systems [13]. For example, the international HSG from the World Health Organization (WHO) on task shifting addresses challenges related to critical workforce shortages, particularly in the area of maternal and newborn health in low-income countries [14]. This HSG recommends a more rational distribution of tasks and responsibilities among cadres of health workers as a strategy for improving access and cost-effectiveness within health systems. It focuses on essential components of the intervention (e.g., training of lay health workers), related actions (e.g., adaptations in task distributions), implementation issues (e.g., preferences), and the implications across other health system components (e.g., adaptations to the health information sub-system that may be needed to capture the tasks undertaken by such workers).

While HSG have led efforts to support low- and middle-income countries (LMIC), system-level guidance has increasingly been seen in higher-income countries too. Cancer Care Ontario (CCO), an advisory body to the province of Ontario, Canada on matters related to quality in cancer, has recently extended its reach to include a more systems-level perspective. CCO has developed several guidance documents designed to inform the organization and delivery of cancer services. For example, its Models of Systemic Therapy guidance document recommends a four-level provincial system to optimize access and decrease wait times to chemotherapy agents [15]. The guidance delineates at each level the clinician team phenotype, institutional phenotype, equipment needs, and safety requirements as a function of the complexity of the treatments and the context in which care will be delivered (e.g., rural versus urban).

Thus, HSG recommendations can help to determine appropriate ways to frame the problem of a population not having access to a primary care physician (e.g., supply, distribution, or payment problem), to outline viable options for health system arrangements that will strengthen primary care (e.g., financial and governance arrangements), to identify alternative implementation strategies that will get cost-effective programs, services, and drugs to those who need them, to monitor implementation efforts, and to evaluate their impacts [16].

There are existing tools to support health systems. For example, while health system performance assessments (HSPA) report on the nature of a specific health problem, help prioritize topics, and evaluate achievements and progress towards HSG goals and recommendations [17, 18], HSG is uniquely positioned to provide specific guidance to help solve a problem and to define the direction to which improvements can be made. The quality of HSG may, therefore, impact the type of recommendations being formulated, the degree to which they get implemented, the methods of dissemination, and the extent to which they impact the usual operations of the health system [19]. Higher quality guidance has the capacity to contribute to higher quality policy decisions [20, 21] which in turn will better optimize health impacts through well-functioning health systems [22].

Clinical practice guidelines (CPGs)—guidance documents that target clinical questions and provide recommendations relevant to (primarily) clinician and patient decisions—could be considered conceptually equivalent knowledge tools to HSG. Both want to advance quality and improve outcomes, use relevant evidence appropriately, and ensure engagement of stakeholders to create implementable and sustainable solutions to health challenges. Optimizing health systems is a challenging task to which appropriate guidance can positively contribute, but there are conceptual and methodological issues unique to HSG that have compromised scientific advancement in this area [11]. There is a paucity of methods to direct the HSG development process, there is a lack of an appropriate conceptual model to appraise HSG quality, and there is a dearth of best practice strategies for reporting guidance recommendations. Health system leaders and researchers lack a framework to ensure that optimal HSG are produced and implemented.

In contrast, there have been considerable advancements made regarding the science and practice of CPG development, appraisal, and reporting. For example, the Appraisal of Guidelines for Research and Evaluation (AGREE) II tool [23] is a reliable, valid, and internationally accepted tool used to direct the evaluation of CPGs and to inform development and reporting goals. Given the similarity in the ultimate aim of CPGs and HSGs, tools such as the AGREE II and the methods used to develop it [24, 25] could provide a foundation from which to strengthen the methodological underpinnings of HSGs. As more groups want to rely on the innovation of HSG, coupled with increasing pressures to demonstrate value for money, there has been an international call to action to create a tool and accompanying resources to support their use and ensure that the most valid, credible, and implementable guidance is identified and applied in health systems [13, 20, 26].

In response, we are conducting a multi-stage program of research with the input of international HSG experts in order to create a reliable, valid, and useful tool for the appraisal of HSG that can also be used to support HSG development and reporting. To fully understand the HSG landscape, stage 1 of this research project was a review aimed at generating a candidate list of concepts (items/domains/criteria) that could comprise a potential HSG appraisal tool. We completed the review (using a critical interpretive synthesis—CIS—approach) of the literature in order to identify any published studies that report on existing criteria currently used to describe, differentiate, or test the quality of HSG [27]. It was our expectation that the receptiveness, adoption, and diffusion of HSG recommendations depend on the perception of their quality, and with this review, we aimed to identify those core elements of a good quality HSG. From the CIS, we identified 30 candidate HSG quality criteria (concepts), clustered into 3 domains, and confirmed that no existing evaluation tool (draft or final version) exists for HSG.

Applying international standards of measurement design for item generation, face validation, and reduction [28], the overall goal of this study (stage 2) was twofold. The first goal was to have the intended users of a potential HSG appraisal tool evaluate the importance, value and priority of the 30 candidate concepts and definitions generated from the CIS, identify any missing components, and from these processes, draft a beta version of the tool. The second goal was to test the usability and performance of the beta version of the HSG tool, further test the face validity of the HSG items and their definitions, and test the anticipated value of the information it generates for users. The purpose of this paper is to report on this stage of the program of research.

## Methods

The approach used for this study was two-step structured surveys targeted at international stakeholders in HSG development and implementation processes. Ethics approval was received from the Hamilton Integrated Research Ethics Board (REB#14-334) and financial support received from a peer-review grant by the Canadian Institutes of Health Research.

For the purposes of this study, and with the goal to create a tool which had international and context relevance, we established an international advisory panel (AGREE HS Research team) comprised of researchers with expertise in strengthening health systems. Many of these advisors also had leadership roles in actual health systems (see the “Acknowledgements” section). With respect to the actual study participants, we sought individuals representing each of the six WHO regions and with health system leadership roles from a government policy lens, clinical lens, and/or health administrator lens.

A candidate pool of potential participant was created by three means: authors listed in publicly available HSG documents, attendees from the Global Symposium of Health Systems Research and the Guidelines International Network Symposium, and by individuals nominated by members of the research team. Each candidate participant was tagged by jurisdiction and type of expertise. The selected individuals made up a master list that served as the population from which participants were purposively sampled for both steps 1 and 2.

For both step 1 and step 2, candidate participants from our master list stratified by location and expertise were invited to participate in the surveys. Letters of invitation, describing the studies, were e-mailed to participants to solicit their participation. Individuals who agreed to participate were e-mailed a password-protected unique identifier to log into a Web-based study platform (LimeSurvey®) to complete the structured surveys. We also accommodated the requests of participants who preferred print packages of research materials. Our letters of invitation to participants outlined the purpose of the study, definition of key terms, likely time commitment, the survey process, and the expected output as well as conditions for participating. Periodic reminders were sent out to the invited participants over the study periods. The surveys were initially pilot-tested by some members of the scientific research team as well as some selected health services/systems researchers, to enable refinements prior to their distribution to consenting participants.

### Step 1

During the 4-month study period for step 1, consenting survey participants were asked to evaluate each of the 30 candidate concepts for appropriateness to, relevance to, and priority for health system decisions and HSG. Each candidate concept was accompanied by an operational definition and considerations for scoring. Specifically, for each of the 30 concepts, participants were asked to rate their agreement with the following four key questions (measures):

1. This concept is a defining feature (core component) of HSG.
2. This is an important concept to address in the development process of HSG.
3. This is an important concept in the appraisal of HSG to differentiate between higher and lower quality guidance documents.
4. This is an important concept to be reported in HSG.

A 7-point scale (1, strongly disagree to 7, strongly agree) was used to rate each of the concepts for each of the questions. In addition, participants were provided with the opportunity to suggest refinements and modifications to each of the candidate concepts (i.e., labels, definitions, etc.) and to suggest additional concepts not addressed in the list. Participants were also asked to rate their overall agreement about the need for a high-quality tool aimed to systematically appraise HSG and contribute to HSG development and reporting. Demographic questions that captured the participants’ gender, affiliation/organization, role/expertise, and years of experience were also included in the survey.

Survey responses were downloaded into Microsoft Excel spreadsheets and analyzed using Excel and SPSS. Overall descriptive analyses were calculated for each of the rated concepts (mean, standard deviation (SD), mode, median, and range). Items that 80 % or more of the respondents rated favorably on each of the four measures (between 5 and 7 on the response scale) were maintained. Those that did not meet this threshold were prioritized for discussion. Additional concepts nominated by the participants were reviewed by the scientific team and reworked to align with the style and format of the other candidate concepts. Written feedback was reviewed and a thematic analysis was done. Final decisions regarding the concepts were made through consensus by the core and extended members of the scientific team. The final list of concepts was reformatted to create the beta version (draft) of the HSG appraisal tool.

### Step 2

The emerging beta version (draft) of the HSG tool was comprised of 32 items clustered into 4 domains. During the 3-month study period for step 2, we collected data on stakeholders’ experiences applying the draft tool on existing HSG documents. The object of inquiry for step 2 continued to be the tool and not the HSG documents themselves.

We purposefully chose three WHO HSG documents from the McMaster University Health Forum’s Health System Evidence database (www.healthsystemsevidence.org). We purposively sampled HSG documents to ensure that we had a mix of (1) guidance addressing health system arrangements as the principal focus and addressing health system arrangements indirectly as a way to get the right mix of programs, services, and drugs to those who need them and (2) delivery, financial, and governance arrangements. Multiple participants rated each HSG document; however, the document was not a variable investigated in this study.

Consenting participants were randomly assigned one of the three HSG documents. Participants were asked to (a) review the HSG document to which they were assigned, (b) review the beta version of the HSG appraisal tool, (c) apply the beta version of the HSG appraisal tool to appraise the HSG document to which they were assigned, (d) answer a series of questions about the appraisal process (i.e., feedback), and (e) provide demographic information.

For the application of the tool, participants indicated whether the concept reflected in each item was documented in the HSG being assessed. Each item was accompanied by an operational definition and a binary response scale (yes/no). For this survey, only 30 of the items on the tool were used to appraise the HSG. Two of the items (implementation plan and evaluation plan) were excluded for this exercise as they only refer to the end users and how they can design a detailed implementation and evaluation plan at the local level for their individual contexts.

Subsequent to the rating of the HSG document, participants were asked to rate their overall agreement on the usability of the HSG appraisal tool as an instrument to systematically direct the development of HSG, to direct the appraisal of HSG, and to direct what needs to be reported in HSG (Yes/No/Uncertain response scale). The participants were also asked to rate the usability of the HSG appraisal tool: were the concepts easy to understand, easy to apply, and was the Yes/No scale appropriate? (7-point scale, strongly disagree-strongly agree). The participants were asked to provide any additional comments on the survey process, on the content of the candidate concepts (operational descriptions/definitions) presented, and on the HSG appraisal tool (perceptions of its usefulness, appropriateness, ease of application). Demographic questions that captured the participants’ gender, affiliation/organization, role/position, years of experience, and previous participation in HSG development were also included.

Survey responses were downloaded and analyzed using Microsoft Excel spreadsheets. Appropriate descriptive statistics were calculated for each of the question groups. The HSG appraisal tool scores were calculated (percentages of the Yes/No responses) and compared within and across the HSGs for exploratory purposes only. Usability of the HSG appraisal tool was assessed by calculating the percentages of Yes/No/Uncertain responses for each of the development, reporting, and evaluation metrics. Overall means were calculated on participants’ ratings that the instrument was easy to understand, the instrument was easy to use, and the rating scale was appropriate. We reviewed the qualitative feedback received and performed a thematic analysis. Final decisions regarding the concepts and the generation of a refined HSG appraisal tool were made through consensus of the members of our scientific team.

## Results and discussion

Table 1 shows the demographic details of the survey participants. The total number of participants invited to step 1 (the importance, value, and priority of the thirty candidate concepts) was 50, and the total number of respondents who completed the survey was 41, for a response rate of 82 %. For step 2, 35 invitations to participate in the usability testing of the beta version of the HSG tool were distributed and 26 complete surveys were returned (response rate of 74 %). For both surveys, the majority of the respondents were men. Respondents represented all six World Health Organization regions with the Americas and Europe most represented and the Eastern Mediterranean and Southeast Asia least represented. In terms of expertise, our respondents represented a variety of health system/health policy roles either at national health ministries or international health agencies, and others were health services/systems researchers either within academia or with applied research institutes. Participants’ years of experience in their roles/position ranged from 1 year to over 40 years. For step 2, we additionally collected data on participants’ years of health system experience and this ranged from 2 to 33 years. Two thirds of our respondents in step 2 had not participated in the development of a HSG document.

**Table 1:** Demographic details

Step 1	Step 2
Gender	Gender
Males = 31 (76 %)	Males = 19 (73 %)
Females = 10 (24 %)	Females = 7 (27 %)
WHO health region	WHO health region
Americas = 17 (41 %)	Americas = 6 (23 %)
Europe = 10 (25 %)	Europe = 5 (19 %)
Africa = 7 (17 %)	Africa = 4 (15 %)
Western Pacific = 4 (10 %)	Western Pacific = 4 (15 %)
Eastern Mediterranean = 2 (5 %)	Eastern Mediterranean = 4 (15 %)
Southeast Asia = 1 (3 %)	Southeast Asia = 3 (12 %)
Role/expertise	Role/expertise
Director = 8 (20 %)	Director = 5 (19 %)
Manager = 4 (10 %)	Manager = 3 (12 %)
Technical adviser = 9 (21 %)	Technical adviser = 6 (23 %)
Researcher (academia) = 8 (20 %)	Researcher (academia) = 9 (34 %)
Researcher (applied) = 12 (29 %)	Researcher (applied) = 3 (12 %)
Years of experience	Years of experience
1–4 years = 5 (12 %)	1–4 years = 5 (19 %)
5–9 years = 12 (29 %)	5–9 years = 7 (27 %)
10–19 years = 11 (27 %)	10–19 years = 7 (27 %)
20+ years = 13 (32 %)	20+ years = 7 (27 %)
	Years of health system experience
	1–4 years = 10 (39 %)
	5–9 years = 10 (39 %)
	10–19 years = 2 (7 %)
	20+ years = 4 (15 %)
	Participation in HSG development
	Yes = 9 (34 %)
	No = 17 (66 %)

### Step 1

Table 2 reports the participants’ ratings (mean and standard deviation) for each of the concepts to the four key questions that were asked in the survey for step 1:

**Table 2:** Means and standard deviations for each concept based on the four outcome measures

Concepts	Core (*C*)		Development (*D*)		Appraisal (*A*)		Reporting (*R*)	
	Mean	SD	Mean	SD	Mean	SD	Mean	SD
1. Priority	6.3	0.9	6.3	0.9	5.9	1.0	6.0	0.9
2. Relevant	6.6	0.8	6.5	0.7	6.2	1.1	6.2	0.9
3. Timely	5.8	1.0	6.0	1.1	5.6	1.2	5.8	0.9
4. Comprehensive	6.1	0.9	6.3	0.7	6.1	0.9	6.3	0.6
5. Systematic and transparent	6.5	0.7	6.7	0.4	6.6	0.5	6.6	0.6
6. Evidence-based	6.6	0.6	6.5	0.8	6.6	0.6	6.5	0.9
7. Participatory	6.4	0.9	6.7	0.5	6.6	0.6	6.5	1.0
8. Ethical	6.5	0.6	6.3	0.7	6.2	0.8	6.3	0.8
9. Outcomes oriented	6.3	0.9	6.5	0.8	6.5	0.8	6.4	0.8
10. Interests managed	6.6	0.7	6.7	0.5	6.7	0.5	6.7	0.5
11. Clearly presented	6.3	0.9	6.4	0.8	6.2	0.8	6.3	0.9
12. Defined problem	6.5	0.5	6.5	0.6	6.3	0.9	6.6	0.6
13. Operational options	5.6	1.2	5.9	1.0	5.9	1.0	5.9	1.0
14. Costs	6.0	1.0	6.2	0.8	5.8	1.1	5.9	0.9
15. Resources	6.1	1.0	6.2	1.0	5.9	1.2	6.0	1.0
16. Effectiveness	6.2	1.2	6.3	0.8	6.0	1.1	6.1	1.1
17. Cost-effectiveness	6.2	1.2	6.1	1.0	5.9	1.3	6.1	1.1
18. Benefits/harms weighting	6.2	1.1	6.3	1.0	6.2	1.0	6.1	1.5
19. Dissemination plan	6.3	0.9	6.3	0.9	6.0	1.1	6.2	1.1
20. Process evaluation	6.2	1.1	6.4	0.9	6.1	1.1	6.2	1.1
21. Outcomes/impact evaluation	6.2	0.8	6.4	0.8	6.2	0.9	6.1	1.1
22. Updating plan	6.1	0.9	6.1	0.9	5.9	1.0	5.9	1.0
23. Feasible	6.4	0.6	6.4	0.7	6.5	0.8	6.3	1.1
24. Affordable	5.5	1.4	5.5	1.4	5.3	1.5	5.5	1.4
25. Flexible	5.6	0.9	5.8	0.9	5.4	1.1	5.4	1.1
26. Socio-cultural alignment	5.9	1.0	6.0	1.0	5.8	1.9	5.9	1.0
27. Political alignment	4.9	1.5	5.0	1.4	4.7	1.6	5.0	1.4
28. External alignment	5.6	1.2	5.8	0.9	5.3	1.4	5.6	1.3
29. Transferable	5.6	1.1	5.7	1.1	5.6	1.2	5.7	1.1
30. Sustainable	5.8	1.0	5.8	1.0	5.6	1.3	5.7	1.3

1. Concept is a core component (*C*) of HSG.
2. Concept is important in the development (*D*) of HSG.
3. Concept is important in the appraisal (*A*) of HSG.
4. Concept is important in the reporting (*R*) of HSG.

As can be seen in Table 2, ratings were universally favorable and, for each concept, there was consistency in the mean ratings across the four metrics (i.e., core, development, appraisal, and reporting). For the *core* metric (*C*), mean ratings fell between 4.9 (political alignment) and 6.6 (interests managed, evidence-based, and relevant). For the *development* metric (*D*), mean ratings fell between 5.0 (political alignment) and 6.7 (systematic and transparent, participatory, and interests managed). For the *appraisal* metric (*A*), mean ratings fell between 4.7 (political alignment) and 6.7 (interests managed). And for the *reporting* metric (*R*), mean ratings fell between 5.0 (political alignment) and 6.7 (interests managed). Standard deviations for all the 30 concepts across all the four outcome measures were small, suggesting consistency in responses across participants.

“Political alignment” was the least favorable concept; for two of the measures, *core* and *appraisal*, it did not reach the mean threshold of 5.0, scoring 4.9 and 4.7, respectively. Nonetheless, the members of our scientific team considered that this concept was important, and in view of the fact that it had only missed the threshold slightly, upon deliberation, the final consensus decision was to include it in the tool.

We also recorded an overwhelmingly high and consistent overall mean agreement in relation to the need for a high-quality tool to systematically direct the development of HSG (6.6), to systematically direct the appraisal of HSG (6.6), and to systematically direct the reporting of HSG (6.3). The standard deviations and the ranges recorded were low, and the modes and medians were 7 for all three categories.

Considerable feedback was also provided by the participants regarding refinements and changes to the wordings of the concepts and their descriptions. However, no additional unique items were suggested by the participants. Using the results and feedback from the survey, reconsiderations of the raw data from the review, and a series of meetings with the core and expanded members of the team (*n* = 11), the concept labels and descriptors were refined. Specifically, two of the concepts that emerged from the CIS (costs and resources) were merged to represent one concept (resources). Also, “process evaluation” and “outcomes/impact evaluation” were merged into “assessment plan”. Additionally, two of the concepts were split into two: “updating plan” became “updating plan” and “up-to-date”, while “systematic and transparent” became “systematic” and “transparent”. Additional file 1 shows a table comparing the original labels and the new labels after the refinement process.

**Figure 1:**
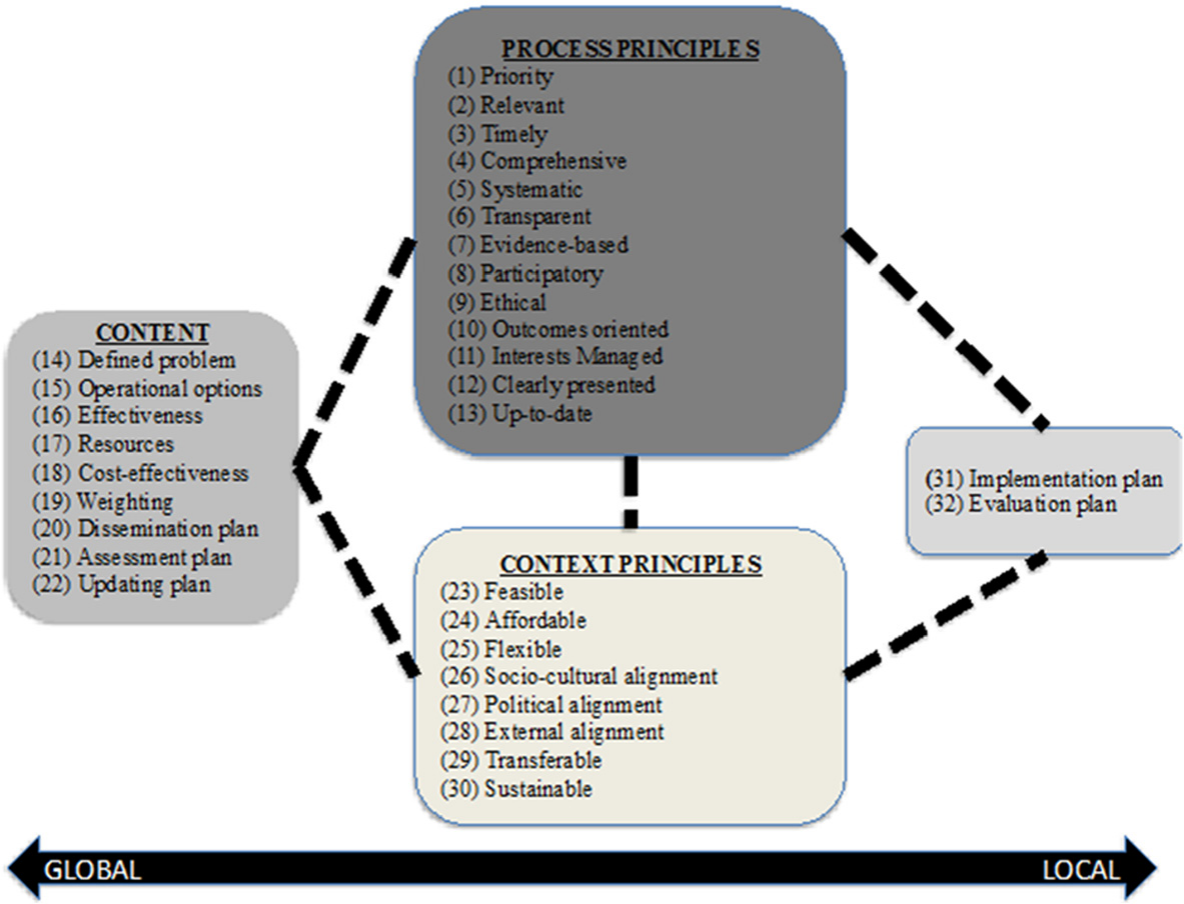
Framework of health systems guidance concepts

The feedback from the survey and deliberations with members of the scientific team also led to the modification of the AGREE-HS framework that shows relationships between the concepts as well as relationships between clusters of the concepts (Fig. 1). Building from our previous study [18], we clustered the concepts together into four meaningful categories (domains): process principles, content principles, context principles, and implementation/evaluation plan. In contrast to the original version of the framework [18], for this version, a double-headed arrow was added to depict the division of labor between roles at the global level and roles at the local level. At the local level, an additional category was added to represent the need for end users to design a detailed implementation and evaluation plan for their individual contexts. The implementation plan represents the development of a strategic plan by the end users to put the guidance recommendations into action. The evaluation plan entails the development of a monitoring and evaluation strategy for the process of implementation as well as the outcomes/impacts of the guidance recommendations. This brought the number of items to a total of 32 clustered into 4 domains.

The beta version of the HSG appraisal tool concepts (labels and definitions), named AGREE Health Systems (AGREE-HS) is presented in Table 3. The beta version of the AGREE-HS was the object of analysis in step 2.

**Table 3:** Beta version of the AGREE for Health Systems (AGREE-HS) tool

Process principles	
1. Priority	The guidance is properly aligned with current health system priorities from the perspective of topic, jurisdictional focus (e.g., all low- and middle-income countries, sub-Saharan Africa), health system level, and population. The expression of the need and origin of the mandate for the guidance is clear.
2. Relevant	The guidance recommendations are relevant to, appropriate to, and valid for the health system challenge, system or sub-system needs, the target population(s), and the setting in which they will operate.
3. Timely	The recommendations are available in a timely manner in relation to when the policy decisions are made or timely in relation to the health system issue being addressed.
4. Comprehensive	The guidance is comprehensive and covers all relevant/appropriate (direct and indirect) health system levels (e.g., district), sub-systems (e.g., mental health), and sectors (e.g., acute care)
5. Systematic	Systematic processes are applied in developing the guidance according to a specific plan and/or explicit methodologies.
6. Transparent	A transparent and reproducible approach in the development and reporting of the guidance is demonstrated.
7. Evidence-based	The best available and ideally most contextually relevant evidence informs the recommendations.
8. Participatory	The health system guidance team is comprised of multidisciplinary/multi-sectoral membership and includes those with an interest, stake, or responsibility in the development, implementation, and evaluation of the recommendations.
9. Ethical	The recommendations are considered within the lens of an ethical framework and align with applicable ethical principles and values (e.g., equity, equality, human rights, liberty, efficiency, autonomy, dignity, beneficence, etc.). The guidance adequately promotes fairness and equality in terms of age, ability, culture, gender, socioeconomic status, religion, occupation, language, ethnicity, race, or sexual orientation among the target population.
10. Outcomes oriented	The guidance describes all the anticipated effects/outcomes as well as the appropriate indicators, performance thresholds, targets, and standards that can be used to measure the effects/outcomes.
11. Interests managed	A declaration of competing interests from the guidance developers (e.g., financial, academic, professional, etc.) is identified and the strategies to manage them are described. It is also clear that the views of any funding body involved have not influenced the development process of the guidance.
12. Clearly presented	The recommendations are clear, user-friendly, succinct, unambiguous, and presented in a readable and consistent format, with key recommendations easily identifiable.
13. Up-to-date	The recommendations are current and the evidence (e.g. systematic reviews) on which they are based is considered up-to-date.
Content	
14. Defined problem	The health system challenge and its causes are clearly articulated; specifically, the nature, causes, and magnitude, frequency or intensity of the problem, and the populations and jurisdictions that are affected are clearly described.
15. Operational options	The recommended “solutions” are operationalized sufficiently with the conceptualization, operational guidance, and the mode of delivery of the options clearly stated.
16. Effectiveness	Evidence of recommendation’s effectiveness are described including methods used, context where tested, and results.
17. Resources	The inputs to and/or the costs of the implementation processes (amounts, frequency, duration) are described and are commensurate to the health systems issue; specifically, money, time, infrastructure, administrative capacity, information, equipment, supplies, health care professionals, training, etc. are considered.
18. Cost-effectiveness	The recommendations are attentive to value for money considerations with relevant cost-effectiveness evidence of recommendations described.
19. Benefits/harms weighting	Descriptions and/or judgments of the potential intended and unintended consequences (positive and negative) of the guidance on the population and/or the system are provided.
20. Dissemination plan	Methods for communicating guidance are clearly described and framed within an overall dissemination strategy.
21. Assessment plan	This involves high-level recommendations for assessing the structure and process of the implementation process as well as an assessment of the outcome/impact of the guidance to determine whether the course of action was a success or failure.
22. Updating plan	Recommendations for periodic updates are made and the procedure to update the guidance is provided with explicit timelines on anticipated review, appropriate expiration date of the guidance, and an explanation of the rationale for the proposed time frames.
Context principles	
23. Feasible	The guidance recommendations are realistic and the actions are pragmatic. The guidance describes facilitators and barriers for implementation.
24. Affordable	The guidance recommendations are affordable within the financial structure and budgetary allocations of the health system.
25. Flexible	The guidance is flexible and adaptable to the expertise of the user and the varying local conditions in the context where implementation will take place.
26. Socio-cultural alignment	The recommendations adopt a socio-cultural perspective and are robust under societal and cultural scrutiny.
27. Political alignment	The political acceptability of the recommendations is considered, and the degree of alignment with political interests and commitments are described.
28. External alignment	Determinants of health system performance that lie outside the formal architecture of the health system but will influence the performance of its functions are considered and described (for example, judicial system, social system, recession, corruption, state of the economy).
29. Transferable	A description of the degree to which recommendations are transferable to other similar or different regions and contexts is provided.
30. Sustainable	The anticipated sustainability and maintenance of long-term outcomes is described.
Implementation and evaluation plan	
31. Implementation plan (end users)	This involves the development of a strategic plan by end users at the local level to describe the process of moving the recommendations into action. The plan may include a description of inputs, services, and activities that are required for implementation; identification of the strengths, weaknesses, opportunities, and threats to the implementation process; and allocation of responsibilities and duties. Designing an implementation strategy will facilitate adherence and compliance to planned activities and enhance efficiency.
32. Evaluation plan (end users)	A strategy for the monitoring and evaluation of the implementation strategy/process and/or outcomes of the guidance in a way that determines whether the changes observed in relation to the health system challenge being addressed can be attributed to the guidance is provided. There are also recommendations for an impact evaluation to look at the short- and long-term deeper primary and secondary changes that resulted from the guidance as well as corresponding challenges.

### Step 2

Table 4 reports quality scores of applying the AGREE-HS on the HSG documents. As can be seen, this exploratory analysis demonstrates quality varied as a function of the AGREE-HS item and varied as a function of the HSG document being evaluated. For example, across the three HSG documents reviewed, higher quality was seen (as reflected with higher percentage of Yes responses) with the AGREE-HS concepts: priority (88, 100, and 100 % for HSG document *X*, *Y*, and *Z*, respectively), relevant (88, 100, and 100 %), timely (88, 90, and 88 %), and defined problem (100, 100, and 100 %). In contrast, lower quality was seen (as reflected with higher percentage of No responses) with the AGREE-HS concepts: cost-effectiveness (12, 30, and 63 % for HSG document *X*, *Y* and *Z*, respectively), assessment plan (25, 10, and 63 %), and external alignment (50, 40, and 12 %).

**Table 4:** Coverage of the AGREE-HS concepts in 3 HSG documents

Concept	HSG Document X^**^		HSG document Y^**^		HSG document Z^**^	
	Yes (%)	No (%)	Yes (%)	No (%)	Yes (%)	No (%)
1. Priority	88	12	100	0	100	0
2. Relevant	88	12	100	0	100	0
3. Timely	88	12	90	10	88	12
4. Comprehensive	37	63	70	30	75	25
5. Systematic	88	12	100	0	75	25
6. Transparent	88	12	90	10	12	88
7. Evidence-based	88	12	100	0	25	75
8. Participatory	88	12	90	10	25	75
9. Ethical	37	63	70	30	75	25
10. Outcomes oriented	37	63	60	40	75	25
11. Interests managed	100	0	80	20	0	100
12. Clearly presented	100	0	100	0	75	25
13. Up-to-date	100	0	90	10	75	25
14. Defined problem	100	0	100	0	100	0
15. Operational options	75	25	70	30	88	12
16. Effectiveness	88	12	70	30	12	88
17. Resources	25	75	30	70	88	12
18. Cost-effectiveness	12	88	30	70	63	37
19. Benefits/harm weighting	88	12	70	30	88	12
20. Dissemination plan	75	25	80	20	25	75
21. Assessment plan	25	75	10	90	63	37
22. Updating plan	63	37	70	30	0	100
23. Feasible	37	63	90	10	100	0
24. Affordable	75	25	70	30	63	37
25. Flexible	88	12	80	20	75	25
26. Socio-cultural alignment	75	25	80	20	75	25
27. Political alignment	100	0	70	30	88	12
28. External alignment	50	50	40	60	12	88
29. Transferable	75	25	80	20	25	75
30. Sustainable	75	25	30	70	88	12
Overall mean %	72	28	74	26	62	38

^**^The object of analysis was not the HSG, so we have denoted them here simply as HSG document X, Y & Z

With respect to AGREE-HS usability measures, participants reported an overall mean value of 5.9 and 5.6, respectively, when asked whether the concepts in the tool were easy to understand and were easy to apply. The standard deviations and the ranges were low, and the modes and medians were both 6 for these two items. In contrast, the mean ratings for the appropriateness of Yes/No scale were less favorable (4.1). Similar to step 1, there was affirmation that the AGREE-HS is a useful knowledge translation tool to systematically direct the development of HSG (85 %), to systematically direct the appraisal of HSG (92 %), and to systematically direct the reporting of HSG (81 %).

We received substantial qualitative feedback from the survey respondents regarding the overall usability of the AGREE-HS tool, suggestions for further refinements, and challenges regarding the Yes/No rating scale that was used. With respect to the latter, participants reported that the dichotomous Yes/No response scale was not appropriate and too constraining for the purposes of evaluation and recommended either a three-item response scale (Yes/No/Partially) or a Likert scale (strongly disagree to strongly agree). Feedback was incorporated into the tool to create the version 1.0 of the AGREE-HS tool (see Additional file 2).

## Conclusions

In step 1 of our study, through a structured survey of relevant stakeholders from all six World Health Organization regions as well as feedback from members of our scientific team, we found that all the candidate concepts for the HSG tool met our a priori criteria for inclusion. Favorable ratings for each item emerged with each of the four target outcomes (i.e., to be included in HSG, to be part of HSG development, to be reported in HSG, and to a criteria on which to rate the quality of HSG). In addition, participants agreed that there was a need for an instrument of this type. These data with feedback led us to refine the HSG framework (Fig. 1) and to create a beta version of the AGREE HS that could be used for testing (Table 3). Together, the data from step 1 provided face validation to our concept of HSG and provided confidence to move the research agenda forward to step 2.

In step 2, participants applied the beta version of the AGREE-HS to assess an HSG and provided feedback on the experience. Our findings showed favorable ratings on the usability of the tool. Items were reported to be easy to understand and easy to use. In contrast, the Yes/No response scale used in the beta version of the tool was not favorably rated. Corroborating findings in step 1, we again found strong support among the participants to create this tool and support for its contents. Finally, we found that in applying the beta version of the AGREE-HS to appraise the three HSG documents, variation in quality emerged between documents and across items, providing preliminary data in its ability to discriminate among HSG reports. Together, the data from step 2 led to refinements to the beta version of the tool. Our final product in this stage of the program of research is the HSG framework and the AGREE-HS version 1.0 (see Additional file 2). It is comprised of 32 items clustered into 4 domains, and each answered with a 5-point response scale (strongly disagree-strongly agree). To our knowledge, this is the first of their kind in the health system research domain.

A key strategy for the production of an acceptable HSG tool is to adhere to standard methodological quality criteria (e.g., usable, reliable, and valid) that confer on guidance the credibility to be used and adapted. This study adds to the existing literature by moving from the generated HSG quality criteria (concepts) to providing a foundation for a knowledge tool and a common analytic framework for health systems that can ultimately improve the HSG enterprise. Given the evidence base upon which the items were generated and two separate studies with knowledge users reporting their favorable support for the concepts, we believe that we have successfully established the face validity of this tool. We believe that this tool will facilitate informed decision-making about HSG at various levels and promoting a culture of informed HSG developers and consumers.

We believe that this tool could be applied by policymakers and health system administrative leaders to differentiate between higher and lower quality HSGs that they might use to inform policy decisions and system redesign. We also believe that these stakeholders could serve as important promoters in elevating the quality of HSG and use of evidence in health system thinking by making the AGREE-HS an expectation among the development community from whom they receive HSG. Developers can use the AGREE-HS as a blue print for their HSG methodological protocols and user manuals with respect to development and reporting expectations. Educators and researchers can use the AGREE-HS as a teaching tool to help learners acquire skills related to health systems.

A strength of this study is that it involved a multidisciplinary blend of international participants recruited based on geography and expertise in order to cover various perspectives and jurisdictions. Secondly, it involved a high-quality approach adapted from the methodological, conceptual, and theoretical principles of measurement construction used to design a complementary tool, AGREE II, which aims to facilitate the development, appraisal, and reporting of clinical practice guidelines. Our methodology was sequential (one step led to the next), differentiated (each step represented a distinctive study required to move to the next step), and cumulative (each step produced data that fed into the overall process). Thirdly, it involved an iterative collaborative process with members of our core and expanded team comprised of investigators and collaborators with an extensive knowledge in health system and policy research. Fourthly, we recruited qualified participants worldwide to ensure that the study resonates with low-, middle-, and high-income countries. Lastly, we asked a wide variety of broad questions that permitted an understanding of the various dimensions of the usefulness of the tool as well as potential areas where issues may arise.

A limitation of this study was that the sample size meant that we did not have sufficient power to also conduct a factor analysis to determine the clustering of items. While not part of the scope of this study, a factor analysis is an important step in the development of a measurement tool [19]. Similarly, and again while not in scope for this stage of the program of research, in a larger sample size, we would have been able to do sub-group analysis to see whether there was any variation in the ratings of the concepts or the ratings of the tool that match directly into specific roles/expertise or jurisdictions. These issues are both being considered for a future study. While it is possible that different results may have emerged with a different sample or a larger sample of participants, the consistency in ratings across participants and small standard deviations give us confidence that our results reflect the perceptions of our targeted communities. Thirdly, while we had an excellent response rate for both steps 1 and 2, we have little information about the demographic characteristics of non-responders and/or the reasons for not responding.

The next steps of our research program involve developing a user manual with more explanations and detailed examples, as well as developing an on-line training program that will be useful for potential users of the tool. We will also proceed with further usability testing, reliability testing, validity testing, and refinement of the AGREE-HS version 1.0 in order to generate the alpha version ready for international unveiling and branding. Of particular interest will be to test its construct validity, its reliability, and its applicability to the various HSGs that exist. We also plan to promote the use of the tool internationally to groups who develop HSG and collate HSG in on-line system directories. The AGREE-HS will join the AGREE family of tools aimed to promote the use of evidence-informed guidance (see www.agreeetrust.org). As we have done with CPGs, our goal is that, through this project, we will contribute to bolstering collaborations among global experts with a wide array of expertise by working towards a common health research goal of creating better quality and more implementable HSG that will improve critical decision-making and lead to stronger health systems for the benefit of patients and populations.

## Additional files

Additional file 1: **Comparison of the original concept labels and the new concept labels.** (DOC 52 kb)

Additional file 2: **Version1.0 of the AGREE for Health Systems (AGREE-HS) tool.** (DOC 55 kb)

## Abbreviations

AGREE-HS: Appraisal of Guidelines for Research and Evaluation for Health Systems
CIS: critical interpretive synthesis
CPG: clinical practice guidelines
DAA: Denis Ako-Arrey
HSG: health system guidance
JL: John Lavis
MB: Melissa Brouwers
MG: Mita Giacomini
SD: standard deviation
WHO: World Health Organization
