# Supplementary material for: Health system guidance appraisal—concept evaluation and usability testing
Source: Implement Sci. 2016 Jan 5;11:3. doi: 10.1186/s13012-015-0365-3 (PMC4700602; doi:10.1186/s13012-015-0365-3)
Supplement: Supplementary file 1 — Comparison of the original concept labels and the new concept labels. (DOC 52 kb) [file 13012_2015_365_MOESM1_ESM.doc]

| **Additional file 1: Comparison of the original concept labels and the new concept labels** | | |
| --- | --- | --- |
| **Original concept labels (CIS)** | **Action** | **New concept label (post survey)** |
| Prioritization | Refined | Priority |
| Relevance | Refined | Relevant |
| Timeliness | Refined | Timely |
| Scope | Changed | Comprehensive |
| Transparency | Split into 2 | Systematic |
|  |  | Transparent |
| Evidence-based | None | Evidence-based |
| Stakeholder involvement | Changed | Participatory |
| Ethical | None | Ethical |
| Outcomes | Refined | Outcomes oriented |
| Competing interests | Refined | Interests managed |
| Presentation | Refined | Clearly presented |
| Problem definition | Refined | Defined problem |
| Operationalization | Changed | Operational options |
| Costs | Merged | Resources |
| Resources |  |  |
| Effectiveness | None | Effectiveness |
| Cost-effectiveness | None | Cost-effectiveness |
| Benefits/harms | Refined | Benefits/harms weighting |
| Dissemination plan | None | Dissemination plan |
| Process evaluation | Merged | Assessment plan |
| Outcomes/impact evaluation |  |  |
| Updating | Split into 2 | Updating plan |
|  |  | Up-to date |
| Feasibility | Refined | Feasible |
| Affordability | Refined | Affordable |
| Flexibility | Refined | Flexible |
| Socio-culturally acceptable | Refined | Socio-cultural alignment |
| Politically sound | Refined | Political alignment |
| External factors | Refined | External alignment |
| Generalizability | Changed | Transferable |
| Sustainability | Refined | Sustainable |
