# Supplementary material for: Health system guidance appraisal—concept evaluation and usability testing
Source: Implement Sci. 2016 Jan 5;11:3. doi: 10.1186/s13012-015-0365-3 (PMC4700602; doi:10.1186/s13012-015-0365-3)
Supplement: Supplementary file 2 — Version1.0 of the AGREE for Health Systems (AGREE-HS) tool. (DOC 55 kb) [file 13012_2015_365_MOESM2_ESM.doc]

| **Additional file 2: Version1.0 of the AGREE for Health Systems (AGREE-HS) tool** | |
| --- | --- |
| **Process principles** | |
| 1. Priority | The guidance is properly aligned with current health system priorities from the perspective of topic, jurisdiction, health system level and population. The expression of the need and origin of the mandate for the guidance is clear. |
| 2. Relevant | The guidance recommendations are relevant to, appropriate to and valid for the health system challenge, system or sub-system needs, the target population(s), and the setting in which they will operate. |
| 3. Timely | The recommendations are available in a timely manner in relation to when the policy decisions are made or timely in relation to the health system issue being addressed. |
| 4. Comprehensive | The guidance is comprehensive and covers all relevant/appropriate (direct and indirect) health system levels (e.g., district), sub-systems (e.g., mental health) and sectors (e.g., acute care) |
| 5. Systematic | Systematic processes are applied in developing the guidance according to a specific plan and/or explicit methodologies. |
| 6. Transparent | A transparent and reproducible approach in the development and reporting of the guidance is demonstrated. |
| 7. Evidence-based | The best available and ideally most contextually relevant evidence informs the recommendations. |
| 8. Participatory | The health system guidance team is comprised of multidisciplinary/multi-sectoral membership and includes those with an interest, stake or responsibility in the development, implementation and evaluation of the recommendations. |
| 9. Ethical | The recommendations are considered within the lens of an ethical framework and align with applicable ethical principles and values (e.g. equity, equality, human rights, liberty, efficiency, autonomy, dignity, beneficence, etc.). The guidance adequately promotes fairness and equality in terms of age, ability, culture, gender, socioeconomic status, religion, occupation, language, ethnicity, race or sexual orientation among the target population. |
| 10. Outcomes oriented | The guidance describes all the anticipated effects/outcomes as well as the appropriate indicators, performance thresholds, targets and standards that can be used to measure the effects/outcomes. |
| 11. Interests managed | A declaration of competing interests from the guidance developers (e.g. financial, academic, professional, etc.) is identified and the strategies to manage them are described. It is also clear that the views of any funding body involved have not influenced the development process of the guidance. |
| 12. Clearly presented | The recommendations are clear, user-friendly, succinct, unambiguous and presented in a readable and consistent format, with key recommendations easily identifiable. |
| 13. Up-to-date | The recommendations are current and the evidence (e.g. systematic reviews) on which they are based is considered up-to-date. |
| **Content** | |
| 14. Defined problem | The health system challenge and its causes are clearly articulated; specifically, the nature, causes, and magnitude, frequency or intensity of the problem, the populations and jurisdictions that are affected are clearly described. |
| 15. Operational options | The recommended "solutions" are operationalized sufficiently with the conceptualization, operational guidance and the mode of delivery of the options clearly stated. |
| 16. Effectiveness | Evidence of recommendation's effectiveness are described including methods used, context where tested, and results. |
| 17. Resources | The inputs to and/or the costs of the implementation processes (amounts, frequency, duration) are described and are commensurate to the health systems issue; specifically, money, time, infrastructure, administrative capacity, information, equipment, supplies, healthcare professionals, training, etc. are considered. |
| 18. Cost-effectiveness | The recommendations are attentive to value for money considerations with relevant cost-effectiveness evidence of recommendations described. |
| 19. Benefits/harm weighting | Descriptions and/or judgments of the potential intended and unintended consequences (positive & negative) of the guidance on the population and/or the system are provided. |
| 20. Dissemination plan | Methods for communicating guidance are clearly described and framed within an overall dissemination strategy. |
| 21. Assessment plan | This involves high-level recommendations for assessing the structure and process of the implementation process as well as an assessment of the outcome/impact of the guidance to determine whether the course of action was a success or failure. |
| 22. Updating plan | Recommendations for periodic updates are made and the procedure to update the guidance is provided with explicit timelines on anticipated review, appropriate expiration date of the guidance and an explanation of the rationale for the proposed time frames. |
| **Context principles** | |
| 23. Feasible | The guidance recommendations are realistic and the actions are pragmatic. The guidance describes facilitators and barriers for implementation. |
| 24. Affordable | The guidance recommendations are affordable within the financial structure and budgetary allocations of the health system. |
| 25. Flexible | The guidance is flexible and adaptable to the expertise of the user and the varying local conditions in the context where implementation will take place. |
| 26. Socio-culturally aligned | The recommendations adopt a socio-cultural perspective and are robust under societal and cultural scrutiny. |
| 27. Political alignment | The political acceptability of the recommendations is considered and the degree of alignment with political interests and commitments are described. |
| 28. External factors | Determinants of health system performance that lie outside the formal architecture of the health system but will influence the performance of its functions are considered and described (for example, judicial system, social system, recession, corruption, state of the economy etc.). |
| 29. Transferable | A description of the degree to which recommendations are transferable to other similar or different regions and contexts is provided. |
| 30. Sustainable | The anticipated sustainability and maintenance of long-term outcomes is described. |
| **Implementation of HSG** | |
| 31. Implementation plan (end-users) | This involves the development of a strategic plan by end-users at the local level to describe the process of moving the recommendations into action. The plan may include a description of inputs, services and activities that are required for implementation; identification of the strengths, weaknesses, opportunities and threats to the implementation process; and allocation of responsibilities and duties. Designing an implementation strategy will facilitate adherence and compliance to planned activities, and enhance efficiency. |
| 32. Evaluation plan (end-users) | A strategy for the monitoring and evaluation of the implementation strategy/process and/or outcomes of the guidance in a way that determines whether the changes observed in relation to the health system challenge being addressed can be attributed to the guidance is provided. There are also recommendations for an impact evaluation to look at the short and long term deeper primary and secondary changes that resulted from the guidance as well as corresponding challenges. |
